# Synaptic Alterations in Mouse Models for Alzheimer Disease—A Special Focus on N-Truncated Abeta 4-42

**DOI:** 10.3390/molecules23040718

**Published:** 2018-03-21

**Authors:** Katharina Dietrich, Yvonne Bouter, Michael Müller, Thomas A. Bayer

**Affiliations:** 1 Division of Molecular Psychiatry, Department of Psychiatry and Psychotherapy, University Medical Center (UMG), Georg-August-University, von-Siebold-Strasse 5, 37075 Göttingen, Germany; katharina.dietrich@medisin.uio.no (K.D.); Yvonne.bouter@med.uni-goettingen.de (Y.B.); 2 Center for Nanoscale Microscopy and Molecular Physiology of the Brain (CNMPB), Humboldtallee 23, 37073 Göttingen, Germany; mmuelle7@gwdg.de; 3 Center for Physiology and Pathophysiology, Institute for Neuro- and Sense Physiology, University Medical Center (UMG), Georg-August-University, Humboldtallee 23, 37073 Göttingen, Germany

**Keywords:** Alzheimer’s disease, N-truncated Aβ, transgenic mouse models, long-term potentiation, electrophysiology, synapse, field potential, Tg4-42

## Abstract

This commentary reviews the role of the Alzheimer amyloid peptide Aβ on basal synaptic transmission, synaptic short-term plasticity, as well as short- and long-term potentiation in transgenic mice, with a special focus on N-terminal truncated Aβ_4-42_. Aβ_4-42_ is highly abundant in the brain of Alzheimer’s disease (AD) patients. It demonstrates increased neurotoxicity compared to full length Aβ, suggesting an important role in the pathogenesis of AD. Transgenic Tg4-42 mice, a model for sporadic AD, express human Aβ_4-42_ in Cornu Ammonis (CA1) neurons, and develop age-dependent hippocampal neuron loss and neurological deficits. In contrast to other transgenic AD mouse models, the Tg4-42 model exhibits synaptic hyperexcitability, altered synaptic short-term plasticity with no alterations in short- and long-term potentiation. The outcomes of this study are discussed in comparison with controversial results from other AD mouse models.

## 1. N-Terminally Truncated Amyloid-β Variants in Alzheimer’s Disease

It is generally well accepted that Alzheimer’s disease (AD) is neuropathologically characterized by extracellular beta-amyloid plaques (Aβ) and neurofibrillary tangles. These pathologies, although typical and important for neuropathological diagnosis of AD, do not convincingly explain synaptic deficits and neuron loss, which are the basis for clinical AD [1]. The amyloid hypothesis was originally based on the discovery that inherited forms of AD can be induced by an enhanced production of full length Aβ peptides [2]. Aβ is released by proteolytic processing of the amyloid precursor protein (APP) [3]. Of interest for the current review is that N-truncated Aβ peptides are major constituents of AD plaques. It was discovered already in 1985 that Aβ (Phe-4; Aβ_4–x_), beginning with phenylalanine at position 4, is a main component of amyloid plaques [4]. Other studies supported the initial findings, and added pyroglutamate Aβ as an additional N-truncated amyloid species [5,6,7,8,9,10], which was previously reviewed in detail [11].

Our group has recently developed a transgenic mouse model for sporadic AD [12]. The Tg4-42 mice express human Aβ_4-42_ and develop an age-dependent massive CA1 pyramidal neuron loss in the hippocampus. The hippocampus-specific expression of Aβ_4-42_ correlated well with spatial reference memory deficits assessed by the Morris water maze test [12,13]. These findings indicate that N-truncated Aβ_4-42_ triggers behavioral deficits comparable to AD-typical memory dysfunction, even without plaque formation and appearance of neurofibrillary tangles.

**Figure 1:**
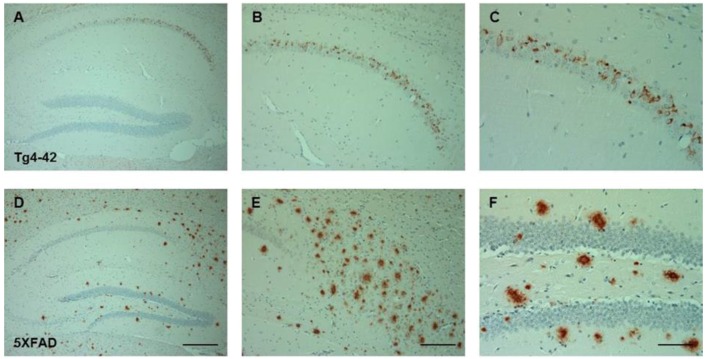
Amyloid pathology in 3-month-old Tg4-42 and 5XFAD. Immunohistochemical staining showing Tg4-42 as an example for an Alzheimer’s disease (AD) mouse model with intraneuronal Aβ and 5XFAD as an example for abundant plaque pathology. Significant intraneuronal Aβ was only detected in CA1 in Tg4-42 mice (**A**–**C**), but not in 5XFAD (**D**–**F**), whereas plaques were only found in the hippocampus of 5XFAD mice. Immunohistochemistry was performed on 4 µm paraffin sections, as previously described [12]. The polyclonal antibody 24311 recognizes pan-Aβ (1:500; rabbit [12]). Biotinylated secondary anti-rabbit and anti-mouse antibodies (1:200) were purchased from DAKO (Glostrup, Denmark). Staining was visualized using the ABC method, with a Vectastain kit (Vector Laboratories, Burlingame, CA, USA) and diaminobenzidine as chromogen. Counterstaining was carried out with hematoxylin (Merck, Darmstadt, Germany). Scale bar: (**A**,**D**) 200 μm; (**B**,**E**) 100 μm; (**C**,**F**) 50 µm.

In order to demonstrate that the Tg4-42 mouse model is a unique AD mouse model, we compared the Aβ pathology to 5XFAD mice [14], a widely used mouse model with typical amyloid plaques. Both models were analyzed with a pan-Aβ antibody in order to visualize intraneuronal Aβ and plaque deposits in CA1 neurons of the hippocampus of 3-month-old transgenic mice. Immunostaining demonstrates strong intraneuronal Aβ accumulation only in the Tg4-42 model (Figure 1A–C), but not in 5XFAD (Figure 1D–F). As expected, 5XFAD mice showed significant extracellular plaque deposition throughout the hippocampus and cortex [14].

## 2. Synaptic Alterations in the Tg4-42 Mouse Model

**Figure 2:**
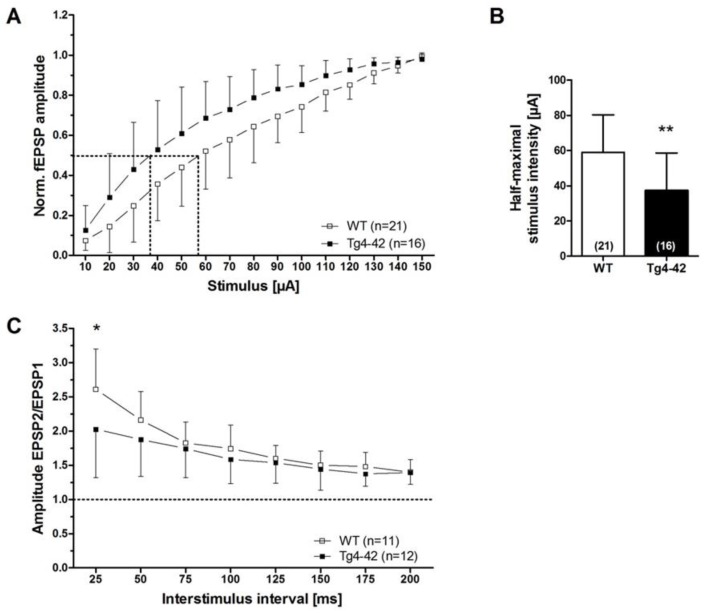
Aβ_4-42_ induced neuronal hyperexcitability and affects short-term plasticity in 3-month-old Tg4-42 mice (taken from [16]). Impact of Aβ_4-42_ on basal synaptic function and short-term plasticity in acute hippocampal tissue slices of Tg4-42 and controls at 3 months of age. (**A**) An altered basal excitatory synaptic transmission was demonstrated by a left shift of the input–output curve; (**B**) The half-maximal stimulus intensity (dashed lines in A) corroborated this observation; (**C**) Paired-pulse facilitation (PPF), quantified as a paradigm for synaptic short-term plasticity, was affected in Tg4-42 mice in comparison to wildtype control mice. (**A** + **C**) Mean ± SD. *n* = number of slices per group (**B**) Mean ± SD. The number of slices analyzed is indicated at the bottom of the bars. Half-maximal stimulus intensity: unpaired *t*-test, ** *p* < 0.01. Amplitude fEPSP2/fEPSP1: unpaired *t*-test, * *p* < 0.05.

**Figure 3:**
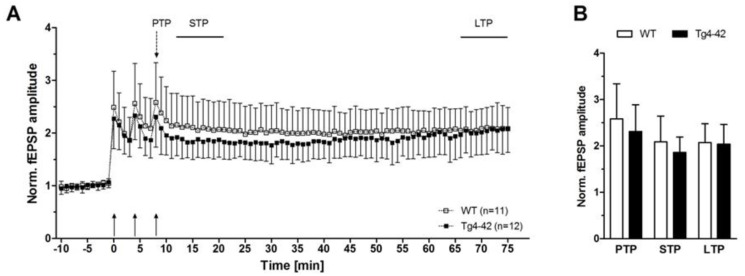
N-truncated Aβ_4-42_ did not alter short-term and long-term plasticity in 3-month-old Tg4-42 mice (taken from [16]). Effects of Aβ_4-42_ on synaptic plasticity were assessed in hippocampal slices of Tg4-42 and wildtype (WT) littermate controls. Post-tetanic potentiation (PTP = was defined as the maximal response within 1 min after the third tetanic stimulus. Short-term potentiation (STP) and long-term potentiation (LTP) were defined as the period between 12th and 21st min, and 65th and 75th min after induction, respectively. (**A** + **B**) Induction of potentiation by trains of high-frequency stimuli triggered PTP, STP, and LTP in both Tg4-42 and control mice. Recordings of STP and LTP revealed stable amplitudes in hippocampal slices of Tg4-42 and WT. Mean ± SD. *n* = number of slices per group.

The decline in synaptic function is an early event in AD pathology. It is mainly related to pathological alterations in the hippocampal formation of AD patients, and correlates well with the clinical symptoms and cognitive dysfunction [15]. Interestingly, expression of Aβ_4-42_ in the CA1 area of the hippocampus in Tg4-42 mice induced certain aspects in synaptic dysfunction and plasticity at a time point prior to neuron death in this model [16]. The main outcomes of this study are summarized as follows. In order to study the possible chronic neurotoxic effects of N-truncated Aβ_4-42_ on synaptic function and plasticity orthodromically evoked field potentials were recorded in hippocampal slices. Field excitatory postsynaptic potentials were evoked at the CA3/CA1 region and orthodromic responses were recorded in the *stratum radiatum* of the CA1 region [16]. Details on the recording conditions were previously published [16]. In short, two slices from each brain of male hemizygous Tg4-42 and wildtype littermate controls (3 months of age) were used (6–8 animals per group). Field excitatory postsynaptic potential (fEPSPs) were evoked by 0.1 ms unipolar stimuli using a steel wire microelectrode. Responses were recorded using glass electrodes [17]. Sampling rate was 20 kHz. The acute hippocampal tissue slices were subjected to three different test paradigms, i.e., input–output curves, paired-pulse facilitation (PPF), as well as recording for short-term (PTP, STP) and long-term potentiation (LTP). Input–output curves were recorded for stimulation intensities of 10–150 µA. fEPSP amplitudes were normalized to their absolute minimum. Four consecutive stimulus trains were pooled and averaged for each stimulus intensity. Remarkably, a left shift of the input–output curve was observed. This is an indication for altered basal excitatory synaptic transmission (Figure 2A). The increased neuronal excitability corroborated this finding with the half-maximal stimulus intensity in 3-month-old Tg4-42 mice (Figure 2B). It has been shown that PPF is a paradigm for synaptic short-term plasticity [18] with a mostly presynaptic origin [19]. Using the half maximal stimulus intensity obtained from input–output recordings, this twin-pulse stimulation was measured at eight different interstimulus intervals (25–200 ms), and calculated as the ratio of the second fEPSP to the first fEPSP amplitude. Recordings revealed characteristic PPF amplitudes declining fast with increasing interstimulus interval duration (Figure 2C). Noticeably, Tg4-42 mice showed lower output intensities—a sign for a decline in short-term plasticity (Figure 2C). Furthermore, the effect of N-truncated Aβ_4-42_ on post-tetanic potentiation (PTP), short-term potentiation (STP), as well as long-term potentiation (LTP) at the Schaffer collateral CA1 pathway was examined. Post-tetanic potentiation is mostly considered to be of presynaptic origin [20], and lasts between 30 s and several minutes. When applying brief high-frequency stimuli trains to the Schaffer collaterals, three different phases can be distinguished: PTP, STP, and LTP. Presynaptic accumulation of Ca^2+^ causes PTP that readily decreases after Ca^2+^ clearance. In this phase, PTP is *N*-methyl-d-aspartate (NMDA) receptor-independent. By contrast, the following two phases (STP, LTP) are of postsynaptic origin, and NMDA receptor-dependent forms of potentiation [21]. Baseline fEPSPs were determined using the half-maximal stimulus intensity and a low stimulation frequency (measured every 15 s; 4× averaged for 1 min) and recorded for 10 min. Different forms of synaptic potentiation were induced by applying three tetanic stimuli, and trains of 100 Hz for 1 s every 5 min. After the third tetanic stimulus, recordings were continued for additional 65 min. Absolute fEPSP amplitudes were normalized to the average of pre-tetanus baseline fEPSP amplitudes. Post-tetanic potentiation was defined as the maximal response within 1 min after the third tetanic stimulus. STP and LTP were defined as the period between 12th and 21st min, and 65th and 75th min after induction, respectively. Induction of synaptic potentiation induced PTP of fEPSP amplitudes with no significant difference between wildtype and Tg4-42 mice (Figure 3A,B). The same was true for STP, which remained stable (Figure 3A,B). Tg4-42 mice showed stable LTP even after 65 min after the high-frequency stimulation (Figure 3A,B). The extent of LTP in Tg4-42 mice was not different compared to wildtype mice.

Therefore, one can conclude that the expression of N-truncated Aβ_4-42_ in the hippocampus of Tg4-42 mice leads to neuronal hyperexcitability, and affects synaptic short-term plasticity, while no significant changes in STP or LTP were observed [16]. This is partially in contrast to previous studies in other AD mouse models. As it is now well established that Aβ_4-42_ oligomers are highly soluble in comparison to full length Aβ_1–42_, we believe that the controversial lack of changes in STP and LTP are due to the different biophysical characteristics of both peptides.

Additionally, distinct Aβ levels might determine synaptic activity in young Tg4-42 mice, as described above. The expression levels of amyloid peptides may influence synaptic activity at the presynaptic site [22]. It is likely that enhanced synaptic excitability could be triggered by the oligomerization state of Aβ. A change in basal synaptic function was recently detected in a mouse model that harbors two FAD-linked mutations. Megill and colleagues found an increase in fEPSP slope and fiber volley amplitude in 2-month-old transgenic mice [23]. Another transgenic mouse model overexpressing mutated APP and mutated Presenilin-1 in neurons also showed hippocampal hyperactivity, as seen in the Tg4-42 model [24]. Previously, Kamenetz et al. observed that activity-dependent Aβ secretion induces a negative feedback loop, thereby influencing neuronal hyperactivity [25]. In good agreement with the outcomes of the Tg4-42 study [16], treating hippocampal CA1 neurons of wildtype mice with nanomolar concentrations of Aβ dimers induced hyperactivity as well [24]. The authors hypothesized that Aβ dimers may induce inward currents, leading to increased firing rate of action potentials and an increase in intracellular Ca^2+^ concentrations [24]. Hippocampal synaptic hyperactivity influences compensatory mechanisms that may be part of network dysfunctions in the hippocampus [26]. These in vitro studies are supported by a report demonstrating that patients with mild cognitive impairment exhibited hyperactivity in the hippocampus/parahippocampal region [27].

## 3. Neurophysiological Alterations in Mouse Models for AD

Numerous other groups found neurophysiological alterations in various mouse models for AD. Progressively more and more studies tried to analyze how amyloid Aβ can affect neuronal and synaptic functioning. For example, several signaling pathways are impaired after receptor binding of Aβ peptides [28]. In the course of this, cellular dysfunction or cell death has been associated with binding of Aβ oligomers to the Frizzled receptor and the low-affinity nerve growth factor. Alternatively, Aβ might be involved in the loss of insulin receptors, bind to prion protein, or interact with cell surface APP, impair kinase activity, impair Ca^2+^ currents at glutamatergic and GABAergic synapses, or directly form pores for Ca^2+^ in the synaptic membrane [28]. Aβ also might affect NMDA receptor functioning like Ca^2+^ homeostasis, oxidative stress, and synapse loss [29,30,31,32], and affect mGluR5 receptor clustering, diffusion properties of mGluR5, and elevated intracellular Ca^2+^ [33]. Aβ might interact with α7 nicotinic acetylcholine receptors, receptor for advanced glycation endproducts, and Ephrin type-B receptor 2 [22]. How Aβ might impair synaptic plasticity is still a matter of scientific debate [34,35].

An overview of neurophysiological alterations in the hippocampus of different mouse models is presented in Table 1, including Tg4-42 and 5XFAD as examples for models with abundant intraneuronal Aβ and/or plaques, respectively.

As the focus of the current review is on similar experimental settings in the hippocampal CA1 region, we did not include other studies on altered synapse functions in other AD mouse models, e.g., PS2APP [66], SAMP8 [67], and PLB1Triple [68]. 

There are obvious discrepancies in the outcomes of the reports. The comparability of studies using mouse models is hampered by several factors: different transgenic expression vectors and promotors, expression levels of transgenes, genetic background of the strains, gender and age of the mice. Many of the AD mouse models develop amyloid plaques, but no or minor neuron loss. The presence of low Aβ levels facilitates the generation of LTP [28,69]. As discussed in detail by K. Dietrich [16], the variations in different methodologies will impact the results of such assays: interface versus submission style recording chambers, differences in the stimulus protocols, variations in the definition of parameters to be analyzed, like the type of normalization of fEPSP, conditions of in vitro preparations, etc.

Most of the listed studies reported a decline of synaptic function, in contrast to the observations in the Tg4-42 mouse model, with a significant increase in synaptic excitability and no impairment in LTP. As mentioned already, the design of the experiments may influence their outcome, which may account for diverging results of studies with the same AD models (Table 1). Reduced synaptic function was often observed in young mice prior to plaque development.

Soluble (and/or intraneuronal) Aβ is a critical key player in AD-related synaptic deficits [70]: The APP E693∆ transgenic model shows oligomerized, accumulated, and intraneuronal Aβ in an age-dependent manner. Aβ plaques develop late, at the age of 24 months. A significant reduction of PPF and LTP was reported in the granular cells of the dentate gyrus, with no effect on basal synaptic transmission. This important report clearly demonstrated that Aβ oligomers trigger synaptic deficits prior to plaque formation [70]. This correlation was further substantiated by studies using other transgenic mouse models, e.g., APPSLPS1 KI [1], 3xTg-AD [64], acrAβ [71], and PD-APP [43]. Terry et al. have reported that only weak correlations exist between psychological values and plaques and tangles, but the density of synaptic markers correlated well in the neocortex of patients with Alzheimer’s disease [72]. The expression levels of synaptic proteins correlated well in AD cases clinically classified by the Clinical Dementia Rating score with more severe cases having a progressive decline [73]. Other studies corroborated these findings, and discuss that intracellular accumulation of amyloid-β is a predictor for synaptic dysfunction and neuron loss in Alzheimer’s disease (reviewed by [1,74,75]). The current study is in good agreement with these observations. 

In addition to the amyloid hypothesis-driven research, there is an increasing interest in gene–environmental interactions. For example, exposure to early life stress has recently been shown to influence synaptic plasticity after induction of epileptic activity [76], and more specifically, early life stress may alter amyloid-β processing and cognition in transgenic Alzheimer mice [77]. 

In summary, the Tg4-42 mice develop early synaptic deficits and neuron loss in the hippocampus, which correlates well with learning and memory dysfunction [12,78]. This is likely due to soluble oligomers of Aβ_4-42_. Of interest, these oligomers are derived from wildtype Aβ sequence and are not mutated as in other studies (cf. [70]). Finally, besides pyroglutamate Aβ_3–42_, Aβ_1–42_, and Aβ_1–40_, Aβ_4-42_ is a major species in the brain of AD patients, and is therefore an important player in the etiology of AD (reviewed in [11]).

## Figures and Tables

**Table 1:** Overview of neurophysiological alterations in hippocampal slices from transgenic Alzheimer mouse models. Electrophysiological recordings of fEPSPs in CA1 subfield (adapted from [16])*.*

Mouse Line (Mutations) (Promoter)	Intra-Neuronal Aβ	Plaques	Input-Output Curve (IO)	PPF	PTP/STP	LTP
Tg4-42 [12] (none) (Thy-1)	>2 m: yes	none	3 m: yes ↑ >12 m: none	3 m: yes ↓ >12 m: none	>3 m: none/ >3 m: none	>3 m: none
TBA2.1hom [36] (Aβ_3E-42_ → Aβ_3Q-42_) (Thy-1.2)	>1 m: yes	>1 m: yes	2 m: none 5 m: yes ↓	n.a.	n.a./ n.a.	2 m: none 5 m: yes ↓
Tg2576 [37,38,39,40,41] (APP: Swe) (PrP)	>2 m: yes	>6 m: yes	2–8 m: none [39,40] 15–17 m: none [40] 12 m: none/yes [41] ↓ 18 m: yes [41] ↓	3 m: none [39] <17 m: none [40] <18 m: none [41]	n.a./ n.a.	3 m: none [39] 2–8 m: none [40] 15–17 m: yes [40] ↓ <18 m: none [41]
PD-APP [42,43] line H6 (APP: Ind) (PDGF-β)	n.a.	2–5 m: none 8–10 m: yes	1–4 m: yes ↓ 8–10 m: yes ↓	1–4 m: n.a. 8–10 m: none	n.a./ n.a.	1–4 m: n.a. 8–10 m: none
PD-APP [42,44] line 109 (APP: Ind) (PDGF-β)	n.a.	27 m: yes	4–5 m: none 27–29 m: yes ↓	4–5 m: yes ↑ 27–29 m: yes ↓	n.a./ n.a.	4–5 m: yes ↓ 27–29 m: none
PD-APP [42,44] line J9 (APP: Ind, Swe) (PDGF-β)	n.a.	2–4 m: none 8–10 m: yes	2–4 m: yes ↓	n.a.	n.a./ n.a.	n.a.
PD-APP [26,45,46] line J20 (APP: Ind, Swe) (PDGF-β)	n.a.	>2 m: yes	3–6 m: yes [45] ↓ 4–7 m: yes [26] ↓	3–6 m: none [45] 4–7 m: none [26]	n.a./ n.a.	3–6 m: yes [45] ↓ 4–7 m: none [26]
APP23 [47,48,49] (APP: Swe) (Thy-1.2)	4 m: yes	>9 m: yes	3–9 m: none 12–18 m: yes ↓ 24 m: none	n.a.	n.a./ n.a.	3 m: none 6 m: yes ↓ 9–12 m: none 18 m: yes ↑ 24 m: none
5XFAD [14,50,51,52,53] (APP: Swe, Flo, Lon, PS1: M146L, L286V) (Thy-1)	>1.5 m: yes	>2 m: yes	4 m: none 5.5 m: yes ↓	<6 m: none	n.a./ n.a.	4 m: none 5.5 m: yes ↓
APPSLPS1KI [54,55,56] (APP: Lon, Swe, PS1: M233T/ L235P) (Thy-1 (APP), PS1 knock-in)	>1.5 m: yes	>2 m: yes	n.a.	2–4 m: n.a. 6 m: yes ↓	n.a./ n.a..	2–4 m: none 6 m: yes ↓
APPswe; PS1∆E9 [23,57,58,59] (APP: Swe, PS1: deltaE9) (PrP)	n.a.	>6 m: yes	6 m: yes [58] ↓ 1 m: yes [23] ↑ 6 m: none [23]	6 m: none [58] 1 m: none [23] 6 m: yes [23] ↓	n.a./ n.a.	6 m: yes [58] ↓ 1 m: yes [23] ↑ 6 m: yes [23] ↓
TgCRND8 [60,61,62,63] (APP: Swe, Ind) (PrP)	n.a.	>3 m: yes	2 m: none [61] 5 m: yes [61] ↓ 6–12 m: yes [62] ↓	2 m: none [61,63] 5–6 m: none [61,63]	n.a./ n.a.	2–5 m: yes [61] ↑ 6–12 m: yes [62] ↓ 2 m: yes [63] ↓ 6 m: yes [63] ↓
3xTg-AD [64,65] (APP: Swe, tau: P301L, PS1: M146V) (Thy-1.2 (APP, tau), PS1 knock-in)	>3 m: yes	>6 m: yes	1 m: none [64] 6 m: yes [64] ↓ 3 m: yes [65] ↓ 8 m: yes [65]↓	1–6 m: none [64] 3 m: yes [65] ↓ 8 m: yes [65] ↓	n.a./ 1–6 m: none [64]	1 m: none [64] 6 m: yes [64] ↓ 3 m: none [65] 8 m: yes [65] ↓

## Abbreviations

The following abbreviations are used in the manuscript:

AD	Alzheimer’s disease
Aβ	Abeta
APP	β-amyloid precursor protein
MALDI-TOF	Matrix-assisted-laser-desorption ionization time-of-flight
pyroGlu-3, AβpE3-x	pyroglutamate Abeta starting with position 3
Asp-1	Abeta starting with aspartate at position 1
Ala-2	Abeta starting with alanine at position 2
Phe-4	Abeta starting with phenylalanine at position 4
Arg-5	Abeta starting with arginine at position 5
fEPSP	field excitatory postsynaptic potential
PPF	paired-pulse facilitation
Ca^2+^	calcium 2+
PTP	post-tetanic potentiation
STP	short-term potentiation
LTP	long-term potentiation
NMDA	*N*-methyl-D-aspartate
RM-ANOVA	repeated measures ANOVA
WT	wildtype
IO curve	input–output curve
PS1	presenilin-1
Swe	Swedish
Flo	Florida
Lon	London
Ind	Indiana
m	age in months
n.a.	not analyzed
↓	decreased
↑	increased
sec	seconds
min	minutes
